# Dietary management of normoalbuminaemic canine chronic enteropathies

**DOI:** 10.1111/jsap.70089

**Published:** 2026-01-25

**Authors:** A. Kathrani, K. Allenspach, D. Dito, J. Hernandez, S. Unterer, M. Vecchio, C. Webb, M. K. Tolbert

**Affiliations:** ^1^ Department of Clinical Science and Services Royal Veterinary College Hertfordshire UK; ^2^ Department of Pathology University of Georgia College of Veterinary Medicine Athens Georgia USA; ^3^ Royal Canin Research Centre Aimargues France; ^4^ Department of Clinical Sciences Nantes‐Atlantic College of Veterinary Medicine and Food Sciences, Oniris VetAgroBio Nantes Nantes France; ^5^ Microbiota Interaction With Human and Animal Team (MIHA) Micalis Institute, Institut National de Recherche pour l’Agriculture, l’Alimentation et l’Environnement, AgroParisTech, Université Paris‐Saclay Jouy‐en‐Josas France; ^6^ Clinic for Small Animal Internal Medicine, Vetsuisse Faculty University of Zurich Zurich Switzerland; ^7^ Clinical Sciences Department, College of Veterinary Medicine & Biomedical Sciences Colorado State University Fort Collins Colorado USA; ^8^ Gastrointestinal Laboratory, Department of Small Animal Clinical Sciences, School of Veterinary Medicine and Biomedical Sciences Texas A&M University College Station Texas USA

## Abstract

Dietary management represents the cornerstone of treatment for chronic enteropathy in dogs, with approximately 50% of cases in referral practice responding to dietary intervention alone. Success rates improve significantly when multiple systematic diet trials are implemented. Clinical experience suggests that dogs with suboptimal response to dietary therapy alone should be maintained on their most effective diet while additional therapies are introduced, potentially reducing medication requirements and associated adverse effects. Treatment decisions should prioritise individual patient assessment and thorough documentation of responses to each dietary intervention rather than adherence to arbitrary trial protocols. Currently, the literature lacks unified nutritional recommendations for canine chronic enteropathy management. This review aims to provide evidence‐based recommendations for the nutritional management of chronic enteropathy in dogs. Recommendations were derived from review of available studies, supplemented by expert clinical experience where published evidence was insufficient. While individual diet selection remains largely guided by empirical evidence, the recommendations presented herein, based on collective clinical expertise, offer a structured approach to optimise therapeutic outcomes in canine chronic enteropathy.

Chronic enteropathy (CE) in dogs involves persistent or intermittent gastrointestinal (GI) signs lasting ≥3 weeks and is diagnosed by exclusion of metabolic, infectious or neoplastic causes. Traditional classification by treatment response [food‐responsive enteropathy (FRE), immunosuppressant‐responsive, or microbiome‐targeted] is misleading, as many dogs do not fit one category and may require different interventions over time (Hodel et al., 2024). Dietary interventions may improve GI tract functions in all types of CE, regardless of treatment classification. Therefore, a comprehensive, individualised nutritional approach (Fig. 1), based on thorough patient evaluation, is essential for all CE cases.

**FIG 1:**
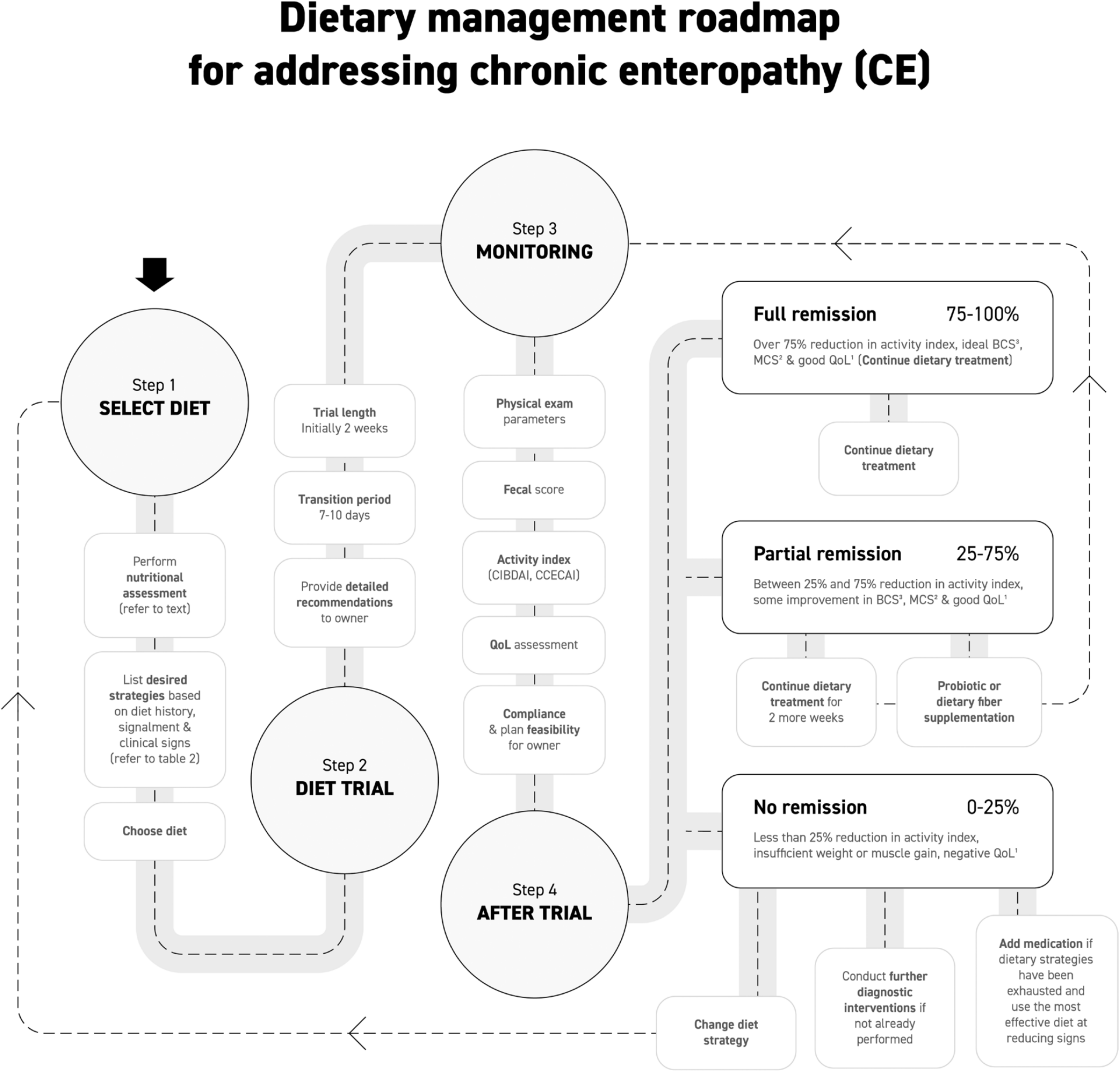
Stepwise dietary management roadmap for dogs with chronic enteropathy. The dietary management approach consists of four main steps: (1) *Select diet* – perform nutritional assessment and choose an appropriate dietary strategy based on diet history, signalment, clinical signs and owner preferences; (2) *Diet trial* – implement chosen diet with a 7‐ to 10‐day transition followed by a 2‐week trial feeding period. Detailed owner recommendations regarding compliance (see Feeding management section in text); (3) *Monitoring* – assess response through physical examination parameters, faecal score, activity indices such as CIBDAI or CCECAI, and quality of life assessment; and (4) *After trial* – evaluate outcomes and adjust management accordingly. Treatment response is categorised as full remission (75% to 100% reduction in activity index with ideal body and muscle condition, and good quality of life), partial remission (25% to 75% reduction in activity index with some improvement in body or muscle condition, and good quality of life) or no remission (0% to 25% reduction in activity index with insufficient weight or muscle gain and negative quality of life). Dogs achieving full remission continue dietary therapy. Dogs with partial remission continue dietary treatment for at least two additional weeks with consideration of probiotic or dietary fibre supplementation. Dogs with no remission should undergo further diagnostic interventions if not already performed, or receive medication if dietary strategies have been exhausted and the most effective diet has been used for reducing clinical signs. Alternative dietary strategies can be trialled at any point if the initial approach is unsuccessful. BCS, body condition score; CIBDAI, canine inflammatory bowel disease activity index; CCECAI, canine chronic enteropathy clinical activity index; MCS, muscle condition score; QoL, quality of life.

Approximately 50% of CE cases respond to diet alone, with higher success rates when multiple trials are attempted (Allenspach et al., 2007; Hodel et al., 2024). Assessing diet‐responsiveness is the most efficient approach to reduce disease severity while avoiding side effects from other treatments (Allenspach et al., 2007; Hodel et al., 2024). When dietary therapy alone is insufficient, the most effective diet should continue alongside non‐dietary treatment to potentially reduce medication dosages and adverse effects. The decision to combine diet with other treatments should be based on individual clinical assessment rather than a predetermined number of diet trials. Thorough documentation of each dietary intervention helps determine optimal treatment combinations.

Food‐responsive enteropathy likely includes immunological adverse food reactions (AFRs) and disorders responding to manipulation of various nutrients beyond protein or nutritional factors (*e.g*. digestibility). Due to this heterogeneous aetiology, achieving remission may require adjustment of multiple nutritional elements.

## INITIAL DIET CHOICE

### Assessing diet and clinical history

Complete dietary history includes all previously and currently fed items: main diet, toppers, treats, medication assistance foods, dental sticks or chews, bones, supplements, preventatives and household food access. Use pre‐appointment forms to streamline collection. Note specific brand name, as nutrient profiles vary dramatically even with similar ingredients. Create a table (Table 1) documenting diets and responses to facilitate an individualised dietary strategy.

**Table 1:** Example dietary history for a canine patient with mixed bowel signs

Diet/Duration	Protein g/100 kcal (specific proteins)	Fat g/100 kcal	Fibre g/100 kcal (specific fibres)	Digestible carbohydrates g/100 kcal	Response
Fresh Pet Beef and Bison/6 months	5.58 GA min (beef, bison, peas)	5.25 GA min	0.65 GA max crude fibre (inulin)	2.3 GA min	Early satiety, vomiting, lip licking, tenesmus, increased frequency of defecation, RC faecal score 5
Purina HA vegetarian/4 weeks	5.26 TNA (soy)	2.65 TNA	1.16 TDF TNA (cellulose, guar gum)	14.93 TNA	Resolved: early satiety, vomiting, lip licking Persistent: tenesmus, increased frequency of defecation, RC faecal score 4 to 4.5
Royal Canin Moderate Calorie PW/7 weeks	7.7 TNA (whitefish, potato)	3.0 TNA	4.7 TDF TNA (cellulose)	13.3 TNA	Rare flares of RC faecal score 4 but otherwise signs resolved

GA Guaranteed analysis, TNA Typical nutrient analysis, TDF Total dietary fibre

Note relationships between clinical signs and daily activities (*e.g*. feeding, exercise). Consider environmental barriers to exclusive feeding in multi‐pet or multi‐person households. For malnourished dogs, have clients measure food in grams, not volume, for accuracy.

### Assessing the patient

Focus physical examination (PE) on oral and dental examination, hydration status, body condition score (BCS), muscle condition score (MCS), rectal exam, skin and hair coat condition, the presence of abdominal pain or masses, and signs of ascites, oedema or intestinal thickening. Ascites or oedema may suggest impaired nutrient absorption and poorer prognosis (Allenspach et al., 2007). Both BCS and MCS are important to note since dogs may experience muscle loss before noticeable BCS changes. Weight loss can significantly impact morbidity and mortality, especially when combined with muscle loss but may be overlooked in previously overweight dogs (Freeman, 2012; Ineson et al., 2019). Skin evaluation is important, as cutaneous signs with large bowel diarrhoea in young dogs suggest food‐responsive disease (Allenspach et al., 2007). After completing the dietary history, PE, and initial diagnostics such as faecal flotation and patient‐specific diagnostics (*e.g*. CBC, serum chemistry, urine cortisol: creatinine, cTLI, serum B12/B9, abdominal ultrasound), identify nutritional strategies for each problem (Table 2). This guides nutrient prioritisation – for example (Table 1), a patient’s upper GI signs may improve with a low‐fat, hydrolysed or novel protein diet, while large bowel signs might require higher total dietary fibre (TDF).

**Table 2:** Conditions and examples of clues that might lead to a better understanding of how to manipulate macronutrients and/or nutritional factors of concern in dogs with CE

Conditions	Clues	Nutritional factor/Nutrient manipulation
Malabsorptive disease	Underconditioned, cachexia, hypocholesterolaemia, flatulence, hypovitaminosis B and D	Consider adjusting protein amount and form (*e.g*. hydrolysed, novel, elemental) or source, increase total digestibility, adjust fat amount and/or source (*e.g*. medium chain triglycerides), vitamin supplementation and adjust feeding frequency/volume
Dietary fat intolerance	Hypocholesterolaemia, steatorrhea, lacteal dilation, pancreatitis, hypercholesterolaemia and hypertriglyceridaemia	Increase total fat digestibility, adjust fat amount and/or source (*e.g*. medium chain triglycerides) and adjust feeding frequency/volume
Adverse food reaction – cutaneous and/or gastrointestinal	Young, pruritus, large bowel diarrhoea ± small bowel diarrhoea or vomiting	Consider adjusting protein amount and form or source (± carbohydrate source), consider increasing EPA/DHA of diet
Fibre‐responsive diarrhoea	Young, large bowel diarrhoea, antibiotic‐responsive diarrhoea	Increase total dietary fibre amount, source and characteristics
Carbohydrate intolerance	Cats>>>dogs, flatulence, bloating, pain, history of eating low carbohydrate diet and switched to higher soluble carbohydrate diet prior to development of diarrhoea	Alter carbohydrate amount, source and digestibility

## ROLE OF INDIVIDUAL NUTRIENTS

### Moisture

Increasing dietary moisture may help reduce dehydration from diarrhoea or vomiting. Higher water intake may protect against the development of CE in dogs and Crohn’s disease in paediatrics (Baron et al., 2005; Trewin & Kathrani, 2023), perhaps because of the effect of water intake on the faecal microbiota composition (Vanhaecke et al., 2022). Although further studies are needed in dogs to determine the therapeutic role of dietary moisture, transitioning to a higher moisture diet could be considered as an additional strategy for CE treatment. However, many canned diets have a different macronutrient profile compared to their extruded counterpart and should be evaluated for their nutritional content before choosing the appropriate option.

### Digestibility

Increasing the total diet digestibility reduces the amount of residual food reaching the colon, which may limit dysbiosis and diarrhoea and lower the production of gut‐derived uraemic toxins from bacterial fermentation of certain amino acids (AAs) (Pilla & Suchodolski, 2021). A highly digestible diet is often defined as one with a protein, fat/carbohydrate digestibility of ≥85% to 87% and ≥90%, respectively. Ingredient selection, processing, cooking and freezing can alter total diet digestibility (Cai et al., 2022). Most veterinary therapeutic GI diets incorporate highly digestible protein sources.

### Fat

Dietary fat modification is a potential therapeutic intervention for dogs with CE. Fat increases energy density, digestibility, palatability and can promote weight gain in underconditioned dogs. In addition, eicosapentaenoic acid and docosahexaenoic fatty acids have anti‐inflammatory and immunomodulatory properties that may be beneficial (Ontsouka et al., 2010). Fat reduction is often needed for dysmotility, hyperlipidaemia, pancreatitis or intestinal lymphangiectasia (Rudinsky et al., 2017; Wennogle et al., 2021) but may result in an increase in required food volume, which can be problematic in animals with a poor appetite or volume intolerance. Determining the appropriate dietary fat content requires assessment of clinical signs and PE findings, concurrent diseases and current dietary intake.

### Protein

Dietary protein provides essential AAs and nitrogen to synthesise non‐essential AAs. Human and porcine data suggest that specific AAs such as glutamine, tryptophan and arginine may play an important role in gut mucosal barrier regulation and immunological function (Abbasi et al., 2024; Tossou et al., 2016). Also, protein and certain AAs increase diet palatability for dogs. Dietary protein can be categorised in several different ways including source (*e.g*. plant, animal and insect), quality (*e.g*. composition, digestibility and bioavailability), amount and form (intact, hydrolysed and elemental). In dogs with a low BCS or MCS, the temptation may be to feed a high‐protein diet. In the presence of a severely diseased small intestine, excess dietary protein may not be readily absorbed leading to osmotic diarrhoea, flatulence and production of gut‐derived uraemic toxins (Ephraim et al., 2020; Lauriola et al., 2023; Zuvarox et al., 2025). Thus, the dietary protein amount needs to be adjusted based on clinical signs and response to the initial diet trial.

### Soluble carbohydrates

Digestion of soluble carbohydrates requires the presence of luminal and intestinal brush border enzymes. Anecdotally, dogs eating a low‐soluble carbohydrate diet such as a raw food diet over a prolonged time may have downregulation of disaccharidase activity and develop diarrhoea when quickly transitioned to diets with high‐soluble carbohydrate. Dogs with CE can develop diarrhoea with diets containing a high proportion of carbohydrates, especially those containing improperly cooked or resistant starches (*i.e*. non‐digestible, fermentable carbohydrates). The type of soluble carbohydrate source and its form can also affect faecal consistency, with individual responses varying based on the dog’s size and breed (Arendt et al., 2014; Weber et al., 2017).

### Fibres

Fibres are predominantly carbohydrate compounds that are resistant to digestion and absorption. Fibre’s effects vary according to their characteristics and the dog size and breed (Weber et al., 2017). Fibres are characterised according to their solubility, viscosity and intestinal fermentability. Bacterial fibre fermentation often results in the production of short‐chain fatty acids, which confer benefits including supporting commensal bacterial growth and activity, inhibiting pathobiont growth, providing an energy source for colonocytes and potentially improving intestinal barrier function (Fritsch et al. 2022a). These advantages may result in several benefits including immune system stimulation, vitamin production, creation of an anti‐inflammatory environment, increased tissue sensitivity to insulin, stool bulking, increased colonocyte absorptive capacity and reduction of toxic metabolites. Soluble, viscous fibres can slow GI transit time and intestinal nutrient absorption (Burkhalter et al., 2001). However, they may also have an AA sparing effect wherein they can conserve AAs by utilising short‐chain fatty acids for energy, allowing proteins to be used for building and repairing tissues (Wambacq et al., 2016). Soluble fibres that are slowly fermentable to non‐fermentable (*e.g*. psyllium) tend to retain their viscosity throughout the intestinal tract, providing a laxative effect for dogs with constipation and a water‐holding, stool bulking effect for those with diarrhoea. Rapidly fermentable, soluble fibres (*e.g*. oligosaccharides) quickly lose this effect in the small intestine. Insoluble fibre (*e.g*. cellulose) is used as a bulking agent to provide a laxative effect and can hasten intestinal transit time. Fibre’s benefits must be weighed with their potential disadvantages including satiety, decreased nutrient digestibility and delayed gastric emptying with viscous fibres. Thus, the TDF, fibre types and the soluble to insoluble fibre ratio must be adjusted to meet the needs of the individual patient.

### Vitamins

Dogs with CE are at risk for micronutrient deficiencies, especially vitamins B and D (Allenspach et al., 2007; Ullal et al., 2023). Cobalamin deficiency can exacerbate intestinal malabsorption and further worsen the cobalamin deficiency (Forshaw, 1969). Reported clinical signs in dogs with hereditary cobalamin deficiency include lethargy, dysorexia and weight loss (Fyfe et al., 1991). Correction of hypocobalaminaemia alongside dietary intervention is associated with a more rapid improvement in clinical signs in cats (Ruaux et al., 2005) and likely in dogs. Hypocobalaminaemic dogs often gain weight and have lessened disease severity when receiving cobalamin supplementation; therefore, blood cobalamin and/or methylmalonic acid concentrations, the latter of which reflects intracellular cobalamin stores, should be assessed and corrected if deficient. Reports of clinical improvement with folate supplementation in CE dogs are lacking; however, anecdotally, folate deficiency can negatively impact appetite and body weight. The prevalence of other vitamin B deficiencies in dogs with CE is underexplored but should be investigated. Treatment with vitamin B complex in hospitalised CE dogs with inadequate food intake or malnutrition is recommended. Vitamin D plays several roles in the body including the maintenance of GI health through regulation of tight junction proteins and inhibition of intestinal epithelial cell apoptosis. As dogs do not synthesise vitamin D in their skin and are reliant on intestinal absorption, they are particularly at risk for vitamin D deficiency with GI diseases. Hypovitaminosis D tends to correlate with disease activity and histological disease severity and is a negative prognostic indicator in dogs with CE (Titmarsh et al., 2015). The benefit of vitamin D supplementation, including an optimal dosing schedule in dogs with CE is currently unknown. Vitamin D supplementation improves clinical and biochemical disease activity in people with inflammatory bowel disease (IBD) (Fabisiak et al., 2017).

### Minerals

Zinc and iron deficiencies are frequently observed in people with IBD (Ghishan & Kiela, 2017). Assessment of total body iron and zinc status can be challenging. In one of the only studies to evaluate iron status in dogs with CE, alterations outside of the reference range for serum iron, serum ferritin and total iron binding capacity were not common, although serum iron and total iron binding capacity did increase post‐treatment (Marchetti et al., 2010). Hypozincaemia may be a common finding in dogs with CE, although assessment of total body zinc is difficult (Sakai et al., 2020). The benefit of zinc and iron supplementation beyond that provided in the diet to dogs with CE without overt signs of deficiency such as cutaneous lesions and anaemia is unknown, and the authors’ do not routinely supplement in these cases. Ionised hypocalcaemia and hypomagnesaemia are also relatively common in dogs with CE. In a large retrospective study evaluating all causes of hypocalcaemia and hypomagnesaemia, dogs with GI disease accounted for 22% and 38% of the cases, respectively (Woods et al., 2021). As hypomagnesaemia may result in a blunted parathyroid hormone response, intravenous correction of total hypomagnesaemia in addition to calcium and vitamin D supplementation is recommended to help improve serum ionised calcium in susceptible patients. Oral administration of magnesium can cause diarrhoea, and therefore, slow dose escalation and close monitoring is required.

## THERAPEUTIC DIET CATEGORIES

Therapeutic GI diets typically have higher digestibility, contain bioavailable intact commonly fed protein sources, highly digestible starch sources and increased levels of sodium and potassium.

This dietary profile has many advantageous effects in dogs with CE (Table 3).

**Table 3:** Advantageous effects of therapeutic gastrointestinal diets in dogs with CE

Strategy	Advantageous effects
Higher digestibility	The higher digestibility results in more rapid dietary protein digestion and absorption in the proximal small intestine and less intact protein, which may decrease stimulation of a dysregulated GI immune system in dogs with CE (Siel et al. 2022)
Restore nutrient absorption	CE often causes inflammatory and morphological changes in the digestive tract, which compromises nutrient absorption (Washabau et al. 2010), and therefore feeding a diet with higher digestibility may counteract this
Increased nutrient absorption	Better absorption of macronutrients in the upper small intestine means less undigested fat and protein entering the colon, which can help restore intestinal eubiosis by limiting the production of microbiota‐derived toxins, and thus reduce worsening of diarrhoea (Devkota et al., 2012; Ramakrishna et al. 1994)
Counter electrolyte abnormalities	Since 19% and 14% of dogs with CE are hypokalaemic and hyponatraemic, respectively (Heilmann et al. 2022), feeding diets higher in these minerals may help to replenish these levels and reduce any associated negative effects

A limited number of published studies have assessed the clinical efficacy of therapeutic GI diets in dogs with CE. In one study, a therapeutic GI diet significantly reduced disease severity from a median pretreatment score of severe to a post‐treatment score of clinically insignificant. However, there was no long‐term follow‐up, as the median time from the start of the diet trial to re‐evaluation was only 13 days (range of 8 to 37 days), and the risk of relapse was not reported (Tornqvist‐Johnsen et al., 2020). In another study, a therapeutic GI diet induced remission, but these dogs were less likely to remain asymptomatic at subsequent rechecks when compared to dogs fed a hydrolysed diet (Mandigers et al., 2010). This category of diets can be considered for those dogs that will not consume a hydrolysed diet and a novel protein diet is not an option. If the dog attains remission with a therapeutic GI diet, transitioning to a hydrolysed diet can be considered to increase the chances of achieving long‐term remission.

Therapeutic GI low‐fat diets have the same attributes as therapeutic GI diets but also incorporate lower fat (typically ≤ 2 to 3 g/100 kcal). High‐fat diets can negatively impact the intestinal microbiota, GI permeability, the mucosal immune response in susceptible animals (de Wit et al., 2012; Devkota et al., 2012; Martinez‐Medina et al., 2014; Miranda et al., 2024; Murphy et al., 2015) and delay gastric emptying (Feinle et al., 1996). Some dogs with CE, especially those with signs of fat malabsorption (*i.e*. steatorrhoea), pancreatitis or dysmotility may benefit from a low‐fat, highly digestible diet. In one of the only studies to specifically assess the effects of a low‐fat GI diet fed to dogs with normoalbuminaemic CE, the response rate (55%) was similar to studies evaluating the effect of a hydrolysed diet (Hernández, 2024).

Therapeutic fibre‐enriched diets are specifically formulated to contain higher fibre, using a mix of soluble, insoluble and prebiotic fibres. Typically, these diets provide ~4.5 to 6.5 g of TDF per 100 kcal, which is relatively lower in TDF and insoluble fibre compared to therapeutic weight loss diets. Due to the many beneficial effects of dietary fibre on the GI tract, as described above, these diets are typically trialled first for those CE dogs with predominantly or exclusively large intestinal signs.

A handful of studies have assessed the efficacy of dietary fibre for the management of canine chronic colitis (Table 4). Overall, the likelihood of response to a fibre‐enriched diet is high, with a response rate of 96% in one study (Leib, 2000) and 68% in another (Fritsch, Wernimont, et al. 2022). The fibre‐enriched diet was safe and well tolerated (Fritsch, Wernimont, et al. 2022), with a mean response time of 8.5 days in one study (Rossi et al., 2020) and within 1 day in another (Fritsch, Wernimont, et al. 2022). One study suggested that long‐term use of the fibre‐enriched diet may be needed to ensure control of the clinical signs (Leib, 2000).

**Table 4:** Studies that have assessed the effect of dietary fibre in canine chronic colitis and their pertinent findings

Study authors	Study design	Pertinent findings
Leib et al. (2000)	Retrospective, uncontrolled	Treatment with a commercial therapeutic highly digestible GI diet supplemented with a median initial dose of two tablespoons of psyllium using the commercial product Metamucil per day resulted in a very good to excellent response in most dogs with chronic idiopathic large bowel diarrhoea For some dogs, the fibre dosage was reduced or eliminated, or a grocery store brand of dog food was substituted, without relapse of the diarrhoea. However, for some dogs, recurrence of signs was seen and therefore owners should be counselled that long‐term fibre enrichment may be warranted
Lecoindre et al. (2011)	Retrospective, uncontrolled	Nineteen dogs with chronic colitis that had failed a low‐fat GI diet responded to different fibre‐enriched diets and maintenance could be achieved without the need for additional medications
Segarra et al. (2016)	Double‐blinded, randomised, placebo‐controlled trial	Assessed the effects of chondroitin sulfate and several prebiotics including resistant starch, beta‐glucan and mannanoligosaccharides in 27 dogs with CE and showed that the histological score of dogs that received the supplement together with a hydrolysed diet decreased by 1.53‐fold, whereas the unsupplemented group showed no difference in histological score. However, a significant difference was not detectable in the canine disease severity score between both groups after treatment, likely due to the small sample size
Rossi et al. (2000)	Prospective, uncontrolled	A high‐fibre, highly digestible, hydrolysed fish diet with a probiotic mixture in 30 dogs with chronic colitis induced the resolution of clinical signs within a mean of 8.5 days (range 4 to 15 days) without additional treatments or the further addition of dietary fibre; however, it is unknown if this effect was due to the diet, probiotic or both
Fritsch et al. (2022)	Prospective, uncontrolled	Investigated the effect of a dry fibre‐enriched (TDF 4.9 g/100 kcal) therapeutic food containing select dietary plant fibres known to contain antioxidant and polyphenol compounds on clinical signs in 31 dogs with chronic large bowel diarrhoea Assessments of overall clinical response and stool parameters indicated that diarrhoea improved significantly within 1 day of initiating the therapeutic diet with 68% of dogs having complete remission of clinical signs by day 56 and the remaining 32% improving with no recurrence Given, the potential disadvantages of increased dietary fibre, including reduction of nutrient digestibility and delayed gastric emptying with viscous fibres, this study importantly showed that the therapeutic food was safe and well tolerated

Therapeutic hydrolysed diets contain peptides derived from chemically or enzymatically treated proteins that are small enough to theoretically avoid a type 1 hypersensitivity reaction by preventing cross‐linking of two immunoglobulin E antibody receptors on a mast cell. Some peptides may retain that antigenic potential, as one study that demonstrated a significant proportion (21%) of dogs sensitised to the intact protein developed cutaneous signs with the hydrolysed protein (Jackson et al., 2003). However, this category of diets has additional attributes beyond their perceived reduced antigenicity that are likely helpful in the attainment of remission in dogs with CE. These include increased digestibility, low fat in some, the use of immunomodulatory ingredients (*e.g*. soy or omega 3 fatty acid) or vegetarian or gluten‐free formulations. The latter two strategies have been shown to be effective in maintaining remission or improving clinical signs in people with IBD, respectively (Chiba et al., 2010; Herfarth et al., 2014).

The evidence for the benefit of hydrolysed diets for the treatment of canine CE is the most robust (Allenspach et al., 2016; Mandigers et al., 2010; Marks et al., 2002; Simpson et al., 2023; Walker et al., 2013; Wang et al., 2019). In one retrospective study assessing long‐term outcome in 203 dogs with CE, where diets were chosen by the attending clinician, 64% of dogs were food‐responsive, with 55% of those dogs responding to a novel protein diet, 44% to a hydrolysed diet and 1% to a home‐cooked diet (Allenspach et al., 2016). In another study, 69% of dogs (20/29) entered remission with a marked reduction in clinical disease scores within 2 weeks of feeding a hydrolysed soy and poultry liver diet (Wang et al., 2019). One controlled prospective study assessed the effects of feeding a hydrolysed soy and poultry liver diet in 18 dogs with CE evaluated over 3 time points during a 3‐year period (Mandigers et al., 2010). At a median 90‐day time point, 89% of dogs responded. At a median of 232 days, 87% were asymptomatic. Finally, at a median follow‐up of 1284 days, of the 14 dogs still receiving the hydrolysed diet, only one had occasional GI signs and two were asymptomatic on the test diet but did develop clinical signs if additional food materials were consumed.

The exact mechanism for the effectiveness of hydrolysed diets is unknown; however, there is some evidence for an immunomodulatory effect (Kathrani & Hall, 2019), as well as a direct effect on the intestinal histopathology and the intestinal epithelial barrier (Marks et al., 2002; Walker et al., 2013). An additional study showed improved microbiota community structure, an expansion of bile acid producing *Peptacetobacter (Clostridium) hiranonis* and improvement in bile acid conversion (Wang et al., 2019). These results highlight the ability of hydrolysed diets to improve or resolve perturbations seen in CE, thereby bringing about remission.

Therapeutic novel protein limited ingredient diets contain a single protein source, which is used less in maintenance diets and is therefore more likely to not have been previously fed to the dog. These therapeutic diets are produced on dedicated equipment to reduce the chances of cross contact of ingredients. This contrasts with non‐therapeutic, over‐the‐counter novel protein diets, which often contain cross‐contacts of commonly fed ingredients due to poor quality control procedures and therefore should be avoided (Raditic et al., 2011). Therapeutic novel protein diets are typically successful for those cases with a suspected AFR because they utilise protein sources that the animal should not have encountered before. This lack of prior exposure means the dog has not developed an immune response to the protein source. Choosing an appropriate diet in this category requires that the veterinarian compiles a comprehensive list of proteins the dog has been exposed to by reviewing the ingredients from all current and previously fed diets, treats and flavoured supplements; however, this may not always be known for the animal. For those cases where a non‐allergic aetiology is suspected, these diets may still be effective due to their limited number of ingredients, increased palatability in some cases and possible differences in moisture, fat, protein, soluble carbohydrate and fibre content.

Novel protein limited‐ingredient therapeutic diets have been extensively studied in dogs with CE (Allenspach et al., 2006, 2007; Gaschen et al., 2008; Luckschander et al., 2006, 2010; Schreiner et al., 2008). One prospective treatment trial in 70 dogs with CE showed 56% responded favourably to a salmon and rice‐based elimination diet. All 39 of these dogs were switched back to their original diet after 14 weeks of the elimination diet, with 31 dogs having no recurrence of original signs for up to 3 years of follow‐up (Allenspach et al., 2007). In another study, a trial with the same diet fed to 51 dogs with CE showed a response in 30 of these dogs (Gaschen et al., 2008). Another study assessing this same diet fed to 29 dogs with CE demonstrated that 19 responded favourably, despite there being no change in the mean histological grade of intestinal biopsy specimens following dietary treatment (Allenspach et al., 2006).

Two separate studies assessed a limited‐ingredient salmon and rice diet at the 10‐day point to improve owner compliance with exclusive feeding. Both studies showed a positive response of 10/26 (38%) and 39/65 (60%) of dogs with CE, respectively (Luckschander et al., 2006, 2010). Further studies assessing a broader range of novel ingredient‐based therapeutic diets, as well as those that combine dietary strategies (*e.g*. low fat, fibre‐enriched), would be beneficial in dogs with CE.

Research on canine CE has shown promise for a benefit of both therapeutic hydrolysed and novel protein diets. This has sparked interest in comparing their efficacy. Two studies, one retrospective and one prospective, found no significant difference in outcomes between dogs fed hydrolysed diets and those fed a limited ingredient novel protein diet or common protein source (fish and chicken) diets (Allenspach et al., 2016; Simpson et al., 2023). However, another retrospective study reported significant clinical improvement in dogs with CE fed a hydrolysed diet compared to a novel protein diet (Marchesi et al., 2017). A randomised blinded controlled trial involving 23 dogs with normoalbuminaemic CE compared three diets: hydrolysed fish with prebiotics, turmeric and high cobalamin, hydrolysed fish without supplements and a control diet of intact chicken and fish. Remarkably, 83% of dogs showed clinical improvement after just 2 weeks, regardless of diet type (Simpson et al., 2023). This suggests that overall dietary change itself, rather than specific protein sources or supplementation, may be key in inducing long‐term remission in some dogs with CE.

Therapeutic elemental protein diets provide protein in the form of individual AAs as opposed to peptides that are found in hydrolysed diets or intact proteins as found in novel protein diets. Other components such as carbohydrate, fat, vitamins and minerals are added to ensure the diet is complete and balanced. The main theoretical benefits of these diets in CE are the elimination of antigenicity and their ease of assimilation due to the use of single AAs. To date, only one study has assessed a commercially available therapeutic veterinary elemental protein diet in dogs with CE (Manchester et al., 2023). In this diet, the protein source but not the fat or carbohydrate is provided in elemental form. Exclusive feeding of the elemental diet improved clinical signs in 16 of 23 dogs with previously uncontrolled CE (Manchester et al., 2023). However, as this was not a controlled study, the comparative efficacy of elemental *versus* hydrolysed diets remains to be determined. Future studies will help to determine the further utility of these diets in dogs with CE.

Home‐prepared diets can be individually designed to target certain desired dietary characteristics such as novel ingredients, low fat, increased digestibility or high fibre. These diets must be formulated using the guidance of a board‐certified veterinary nutritionist to ensure that they are complete and balanced for long‐term feeding (Stockman et al., 2013). The beneficial effects of home‐cooked diets are likely due to their increased digestibility and incorporation of patient‐targeted nutritional strategies. Other theoretical benefits might include the absence of emulsifiers and preservatives, which have been shown to have adverse effects on the intestinal microbiota and GI mucosal immune system in rodent models of IBD (Chassaing et al., 2015; Sandall et al., 2020; Shang et al., 2017; Watt & Marcus, 1971), as well as the avoidance of antigens that may have formed or been unmasked during the manufacturing of commercial pet food (Biourge et al., 2004).

Beyond anecdotal evidence, there is very little published on the use of home‐cooked diets for dogs with CE. Several retrospective studies have sporadically reported dogs that have responded to a home‐cooked diet. In one study, two dogs fed a home‐cooked diet responded, but the source of protein was not disclosed (Allenspach et al., 2016). In another study, two dogs fed a home‐cooked diet responded; one was based on ostrich and the second on chicken (Kawano et al., 2016). Another study assessed the effects of a home‐cooked diet supplemented with coconut oil, rich in medium chain triglycerides, in 18 dogs with CE. All dogs were reported to respond well to the diet change, demonstrating an improvement in their clinical signs following dietary treatment (Vecchiato et al., 2023). Given the scarcity of studies available in the literature assessing home‐cooked diets in dogs with CE, this should be investigated further to define which dogs are likely to benefit from this approach. It is important to note that one study did show that home‐cooked diets cost more than commercially prepared dry kibble diets for dogs with CE (Kratzer et al., 2022).

## FEEDING MANAGEMENT PLAN

### Transition period

A transition period of 7 to 10 days (*i.e*. 10% to 15% new food with 85% to 90% old food on day 1 with continued incremental decreases and increases of the old and new food, respectively) is recommended. For those dogs where signs worsen, slowing down the transition between diets may help to improve the signs, because in the authors’ experience, these tend to be transitory and improve within a few days. For some cases, clinical signs may improve initially during the transition period but then worsen as the proportions of the two diets are further adjusted. If this occurs and does not improve once the dog is fully transitioned onto the new diet, consideration could be given to feeding the proportions of the two diets that resulted in the most improvement in clinical signs if the previous diet is complete and balanced and suitable for the life‐stage of the dog.

### Feeding frequency

Feeding multiple small meals daily may help to improve gastric transit, prevent overloading of the GI tract and therefore limit exacerbation of osmotic diarrhoea, and improve digestion and nutrient absorption. However, the feeding frequency should be assessed for each individual case, because in some instances, dogs may do better when fed less frequently.

### Treats

The new therapeutic diet should be fed exclusively to allow assessment of the chosen dietary strategy. If treats are needed during the trial, then the therapeutic diet can be used or, if available, hydrolysed treats can be fed. If feeding dry food, an allotted amount can be used as treats during the day. If feeding a canned diet, it can be used to make biscuits in the oven to use as treats (*i.e*. sliced loaf of canned food can be moulded into small treats and baked in the oven at 175°C until desired consistency is reached). Although feeding any other types of treats is not ideal, anecdotally dogs can still go into remission if the main therapeutic diet is fed with other foods. Therefore, this approach may be preferable in dogs without AFR than abandoning the trial, if treats are <10% of the daily calories to avoid unbalancing the diet. For those dogs that achieve remission with the therapeutic diet, treats can be introduced after a 6‐ to 8‐week period of exclusive feeding. Ideally, single ingredients should be added one at a time to assess tolerance.

### Length of trial

Response to dietary changes in dogs with CE typically occur within 2 weeks of starting the diet following the transition period (Allenspach et al., 2007; Guilford et al., 2001; Schmitz et al., 2015; Walker et al., 2013). Dogs responding to fibre‐enriched diets often show even quicker responses (Fritsch, Wernimont, et al., 2022; Rossi et al., 2020). Despite the potential for rapid response, the exclusive feeding trial should be maintained for at least 2 weeks before considering alternative therapeutic strategies. If a favourable response is seen, the trial should be continued for a minimum of 6 weeks (Luckschander et al., 2006, 2010; Walker et al., 2013; Wang et al., 2019), or 12 to 13 weeks if concurrent dermatological signs are present (Olivry et al., 2015; Tiffany et al., 2019). If no improvement is seen after 2 weeks of exclusive feeding, transition to another diet, alternative treatment, such as glucocorticoids, or further investigations such as endoscopy or biopsy, if not already performed, can be considered.

## IMPROVING COMPLIANCE

Establishing good owner compliance during diet trials is critical to the success of CE management (Table 5). Animal compliance is also critical for the success of a dietary trial; if the dog does not consistently consume the prescribed diet, the trial becomes uninformative (Table 6). By employing a combination of approaches from Tables 4 and 5, veterinarians can significantly improve the chances of successful dietary trials and, consequently, the management of CE.

**Table 5:** Strategies to increase owner compliance with dietary trials

Strategy	Reason for increasing owner compliance
Stressing importance of diet	Owners should be educated on the importance of diet, emphasising that most cases can be managed with diet for long periods if there is strict adherence
Trial and error approach	Preparing owners for the “trial‐and‐error” approach to finding the best diet for their dog and reminding the owner that the diet trial is a diagnostic tool in this case helps avoid frustration when subsequent diet trials are necessary
Consequences of not following advice	It is important to review the potential consequences of failing to identify the best diet for their dog with the owners, including the use of immunosuppressant medication and their associated side effects, continued or progressive clinical signs and poorer long‐term outcomes
Providing clear dietary recommendations	Supplying the owner with the exact name of the diet, dry or canned formulation, amounts to feed and frequency of meals per day, length of the diet trial, where to source the diet from and addressing their request for treats, if applicable, are important to ensure owners are given all the information they need, so they are less likely to sway from recommendations
Utilisation of veterinary technician or nurse	The veterinary technician or nurse could be empowered to give nutritional recommendations, and the recommendations could be provided as a written hand‐out to reinforce the information
Ensuring all verbal communications are relationship‐centred	By using non‐verbal cues, checking in with the owners’ thoughts and pre‐empting and addressing their worries, as well as signposting, so they know who to contact with any concerns and forward booking recheck appointments in advance will also help to improve owner compliance with diet trials

**Table 6:** Environmental and dietary modifications, and pharmacological interventions to try to improve pet‐related compliance with dietary trials

Environmental and dietary modifications	Pharmacological interventions
Providing the diet on a dinner plate Adjusting mealtimes to coincide with the family meal times to encourage social competition with human family members Cleaning food bowls between meals Feeding in the presence of other dogs may also stimulate social eating behaviour Selecting diets with known palatable ingredients, when possible If above is not possible, utilising diets with novel ingredients may help to stimulate interest and therefore intake Gently warming the food, adding water or using a palatant enhancer, such as a probiotic with animal digest or yeast extract, as a topper may also help to increase acceptance	Can be considered at any time during the diet trial Includes administration of appetite stimulants, management of nausea and controlling pain with appropriate analgesics These medications can gradually be tapered as the diet begins to alleviate the gastrointestinal signs

## ASSESSING EFFICACY OF THE TRIAL

### Monitoring tools

The CE severity should be assessed at baseline and throughout treatment using established disease scoring indices [*i.e*. canine CE clinical activity index (CCECAI) (Allenspach et al., 2007) or the canine inflammatory bowel disease activity index (CIBDAI)] (Jergens et al., 2003). The CCECAI is the summation of nine variables including attitude/activity, appetite, vomiting frequency, stool consistency, defecation frequency, weight loss, blood albumin concentration, and the presence and severity of ascites, peripheral oedema or pruritus. The CCECAI may be superior to the CIBDAI, which omits the presence of ascites and hypoalbuminaemia in its scoring and has also been validated as a risk assessment tool, unlike the CIBDAI (Allenspach et al., 2007). The severity of the activity index at baseline is not necessarily predictive of treatment response; however, it is helpful in the identification of dogs that warrant more intensive diagnostic evaluation and monitoring. Another important monitoring tool would be faecal scoring. Often, clients do not consider their dog as having diarrhoea until the stool consistency is liquid, the dog starts to defecate in the house, or requires nighttime access to outside to defecate. Thus, use of a faecal scoring system at baseline and throughout monitoring can help define a chronic history of diarrhoea that may otherwise go unrecognised by the client. Constipation may occur in some dogs consuming a hydrolysed protein diet due to the low total dietary fibre. If this occurs, a fibre supplement, such as soluble, viscous and slowly fermentable (*e.g*. psyllium), may be used with the diet to alleviate this effect.

### Owner perception and quality of life

Evaluating the quality of life (QoL) including the physical, behavioural/psychological and social well‐being of both the dog and the client helps in supporting the client, evaluating response to treatment and guiding treatment decisions. Although several QoL assessment surveys are available online, none of these have been validated in CE patients. The client’s perception of the dog’s QoL (Diaz‐Reganon et al., 2023; Marchetti et al., 2021; Treese et al., 2021) negatively corresponds with the severity of the dog’s enteropathy (Marchetti et al., 2021). The QoL often improves with treatment regardless of the treatment subcategorisation and therefore offers an additional valuable monitoring tool. The client’s QoL is also negatively affected by their dog’s enteropathy (Diaz‐Reganon et al., 2023). In one study, clients reported symptoms of caregiver burnout, including a reduction in their social life and daily activities. Clients suggested that a more detailed explanation of the disease process and what to expect would have been helpful in lessening the impact of the dog’s disease on the client’s QoL. Strategies to minimise the stress of the caregiver (*e.g*. providing more information about the chronicity of disease, the reality of intermittent flares even with good control, providing at‐home shelf stable strategies for alleviating flares), and therefore likely also the stress on the patient, should be employed.

### Physical examination parameters

Several PE parameters including body weight, body and muscle condition, and coat appearance provide important information about the efficacy of the trial. These parameters should be followed separately as monitoring tools, as these are not included in disease severity scoring systems.

## DEFINING ENDPOINTS AFTER THE TRIAL

### Full remission

One study defined full remission as a reduction in the clinical disease activity index of ≥75% (Jergens et al., 2010). This definition holds regardless of whether the diet was introduced alongside glucocorticoid therapy. The disease activity index, however, does not take into account certain variables, such as weight gain, MCS or QoL factors, which may be important in assessment of response. Recording serial body weights, BCS, MCS and QoL at diagnosis and at defined recheck points will help determine when full remission has been achieved (Fulmer et al., 2022; Lavan et al., 2023; Schmutz et al., 2022).

### Partial remission

Partial remission was defined in one study as a reduction of between 25% and 75% in the clinical disease activity index (Jergens et al., 2010). However, other parameters not covered by the activity index (BCS, MCS and QoL) should also be taken into consideration when determining response. For those dogs exhibiting a partial response to dietary management, the diet could be trialled for a few more weeks, especially if the diet trial is relatively early in its course or additional supplementation with probiotic or dietary fibre could be considered based on the clinical signs. If no response to the original diet trial or additional supplementation is seen following a further 2 weeks, then further diagnostic investigation or a new dietary strategy should be considered. In most cases of otherwise healthy dogs, it should only be after multiple dietary strategies have been exhausted that the addition of medications should be considered.

### Lack of remission or worsening

Lack of remission was defined by one study as achieving <25% reduction in the CCECAI (Jergens et al., 2010). When this occurs, or there is insufficient weight or muscle gain for underconditioned or cachectic patients, respectively, or when owners report poor QoL or worsening of any parameters, clinicians should consider alternative approaches. These may include trying a different dietary strategy, adding medication if dietary strategies have been exhausted or conducting further diagnostic investigations. Other possibilities for failure of signs to improve include owner non‐compliance, concurrent diseases (*e.g*. pancreatitis) or misdiagnosis (*e.g*. alimentary small cell lymphoma).

## TRANSITIONING AFTER A SUCCESSFUL TRIAL

The severity of histopathological disease does not correlate with prognosis. Most dogs with CE, especially younger dogs with milder clinical signs, have a good prognosis and respond readily to dietary intervention (Allenspach et al., 2007). Over time, some dogs that are responsive to diet, but generally not those with AFR, can switch back to their original diet and might continue to be free of clinical signs. In one study, 31 of 39 dogs were able to switch back to their original diet without recrudescence of clinical signs after 14 weeks of the diet trial (Allenspach et al., 2007).

For dogs suspected to have an AFR, a re‐challenge with the suspected offending food antigen(s) can be considered. Relapse within days to 2 weeks of provocation with the previously fed food antigen and improvement following removal of the food antigen confirms the AFR diagnosis. The amount of food antigen required to elicit a reaction in dogs with AFR is likely patient dependent. Dogs with concurrent dermatological signs might be more likely to require strict elimination diets. In a study of dogs with cutaneous AFR, a flare was triggered in two dogs following ingestion of one teaspoon of the previously fed diet (Tinsley et al., 2024). Many owners whose dogs are well managed with a specific diet may be reluctant to change this due to concern for return of clinical signs. Therefore, if elected by the owner, the trialled diet can be fed life‐long if it is complete and balanced for long‐term feeding.

Regardless of the category of CE, it is common for dogs with CE to have symptomatic flares even with a well‐controlled environment and good management practices. Clients should be informed that CE is like other chronic diseases (*e.g*. diabetes mellitus) and that flares should be anticipated. The diet should not be immediately changed because of one flare. Clients should be equipped with a “therapeutic toolbox” containing tools that can be used at home when early signs of flares are evident. Based on comfort level with the client and the dog’s predominant signs, examples of therapies that might be contained in the toolbox include soluble, insoluble or mixed fibre sources, adsorbents, probiotics, synbiotics, anti‐emetics or anti‐spasmodics. Some dogs who initially respond to the diet may develop progressive CE and may require additional or alternative treatment strategies in the future (Hodel et al., 2024). Dogs with AFR that continue to have more frequent flares despite eating an appropriate therapeutic diet should prompt pursuit of a diet change (Masuda et al., 2020). In these cases, it is recommended to keep the dietary characteristics (*i.e*. TDF, fat content, *etc*.) similar to that of the previous diet while changing the suspected offending food antigen(s).

Canine CE represents a complex and multifaceted clinical challenge that demands a nuanced, patient‐specific approach (Fig. 1). The evolving understanding of CE highlights the limitations of traditional categorical classifications and underscores the importance of a comprehensive, adaptive management strategy. By recognising the heterogeneous nature of CE and embracing a holistic approach that emphasises the importance of dietary therapy targeted towards the individual’s needs, veterinarians can optimise patient outcomes. Future research should focus on refining diagnostic techniques, understanding the underlying pathophysiological mechanisms and developing more personalised therapeutic protocols that address the unique characteristics of each individual dog’s GI disorder.

## Author contributions

**A. Kathrani:** conceptualization; writing – review and editing. **K. Allenspach:** conceptualization; writing – review and editing. **D. Dito:** conceptualization; writing – review and editing. **J. Hernandez:** conceptualization; writing – review and editing. **S. Unterer:** conceptualization; writing – review and editing. **C. Vecchio:** conceptualization; writing – review and editing. **C. Webb:** conceptualization; writing – review and editing. **M. K. Tolbert:** conceptualization; writing – review and editing; writing – original draft.

## Conflict of interest

Drs. Kathrani, Allenspach, Hernandez, Webb, Unterer and Tolbert are members of the Royal Canin GI Scientific Advisory Board. Dr. Tolbert has received honoraria and/or paid travel expenses for speaking engagements on behalf of Royal Canin, Hill’s, Nestle Purina, Purina Institute and TikiCat and has received funding from Royal Canin and Nestle Purina for scientific research. Dr. Allenspach has received paid travel expenses from Nestle Purina. Dr. Kathrani has received honoraria from Nestle Purina, Purina Institute, Royal Canin and Hill’s and has received funding from Waltham, Royal Canin and Nestle Purina for scientific research. Dr. Unterer has received honoraria and travel expenses from Nestle Purina and Hill’s. Drs. Hernandez and Tolbert have received funding from Royal Canin for scientific research. Drs. Vecchio and Dito are employees of Royal Canin. Dr Kathrani is an associate editor of the Journal of Small Animal Practice and a co‐author of this article. She was excluded from editorial decision‐making related to the acceptance of this article for publication in this journal.

## Data Availability

Data sharing is not applicable to this article as no datasets were generated or analysed during the current study.
